# Echocardiographic evaluations of right ventriculo–arterial coupling in experimental and clinical pulmonary hypertension

**DOI:** 10.14814/phy2.14322

**Published:** 2019-12-25

**Authors:** David Boulate, Myriam Amsallem, Tatiana Kuznetsova, Roham T. Zamanian, Elie Fadel, Olaf Mercier, Francois Haddad

**Affiliations:** ^1^ Research and Innovation Unit Hôpital Marie Lannelongue Université Paris‐Sud Le Plessis‐Robinson France; ^2^ Division of Cardiovascular Medicine and Cardiovascular Institute Stanford University CA USA; ^3^ Research Unit Hypertension and Cardiovascular Epidemiology KU Leuven Department of Cardiovascular Sciences University of Leuven Flanders Belgium; ^4^ Division of Pulmonary and Critical Care Medicine Vera Moutlon Wall Center for Pulmonary Hypertension Stanford University CA USA

**Keywords:** echocardiography, pulmonary hypertension, right heart, ventriculo–arterial coupling

## Abstract

**Background:**

Tricuspid annular systolic excursion (TAPSE) or velocities (s′) and right ventricular (RV) end‐systolic dimensions are predictors of outcome in patients with pulmonary hypertension (PH). We explored the value of combining peak s′ and RV end‐systolic area index (RVESAi) as a surrogate of RV‐pulmonary artery (RV–PA) coupling in a large animal of precapillary PH as well as clinically.

**Method:**

The first experimental group included four control and four piglets with thromboembolic disease. RV–PA coupling was assessed by ventricular to arterial elastance ratio (Ees/Ea) at baseline, after esmolol and dobutamine administration. Echocardiographic metrics included s′, TAPSE, fractional area change (RVFAC), and RVESAi. The findings were validated in six piglets with severe PH. Clinical cohorts were stable outpatients (*n* = 141) and acutely decompensated pulmonary arterial hypertension (*n* = 48).

**Results:**

In the first experimental group, the best linear correlates of Ees/Ea were s′ (*R* ^2^ = .51, *p* < .001) and RVESAi (*R* ^2^ = .50, *p* < .001), while RVFAC (*R* ^2^ = .17, *p* = .01) and TAPSE showed weaker association (*R* ^2^ = .21, *p* = .39). The ratio s′/RVESAi showed nominally but not significantly (higher) association with Ees/Ea (*R* ^2^ = .58, *p* < .01). The association between changes in s′/RVESAi and Ees/Ea was strong (*R* ^2^ = .56, *p* < .001). In more severe PH, Ees/Ea and changes in Ees/Ea correlated significantly with s′/RVESAi and changes in s′/RVESAi (*R* ^2^ = .69; *p* < .001 and *R* ^2^ = .64, *p* < .001, respectively). In the two clinical cohorts, the s′/RVESAi did not emerge as a stronger predictor of outcome than RVESAi.

**Conclusion:**

RV s′/RVESAi index represents a reasonable bedside‐usable surrogate of RV–PA coupling and of its acute variations in PH. Its incremental prognostic value over end‐systolic dimension alone remains to be proven.

## Graphical abstract

Non invasive evaluation of right ventricle ‐ pulmonary artery coupling may be of experimental and clinical interest. Here we described correlations between simple right ventricular echocardiographic parameters and right ventricule—pulmonary artery coupling measured using the gold standard method (i.e. multibeat pressure‐volume loops). We concluded that peak velocity of the tricuspid annulus (s′), end‐systolic area, as well as there ratio, may represent interesting surrogate of right ventricle ‐ pulmonary artery coupling.

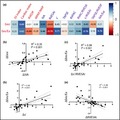

## 1 INTRODUCTION

Right ventricular–pulmonary arterial (RV–PA) coupling quantifies the adaptation of the right ventricle to its afterload. It is considered as a major determinant of functional capacity and survival in patients with precapillary pulmonary hypertension (PH) (Amsallem, Kuznetsova, Hanneman, Denault, & Haddad, 2016; Vanderpool et al., 2015). The gold standard to quantify RV–PA coupling is the ratio of RV end‐systolic elastance (Ees, a load independent index of RV contractility) to pulmonary arterial elastance (Ea, an index of RV afterload) ratio (Dell'Italia & Walsh, 1988). Ees and Ea are measured invasively requiring multibeat RV pressure–volume (PV) loop acquisitions using dedicated right heart catheterization associated with acute preload variation, often induced by transient occlusion of the inferior vena cava (as detailed in the Figure S1 [https://figshare.com/articles/Figure_S1_tif/9946838]) (Boulate et al., 2016). Recently, the simple ratio of RV stroke volume over end‐systolic volume (SV/ESV) measured from magnetic resonance imaging has been proposed as a marker of ventriculo–arterial coupling assuming a zero intercept (Brimioulle et al., 2003; Sanz et al., 2012), and shown to be independently associated with survival in patients with PH (Vanderpool et al., 2015).

The present study sought to investigate whether a similar simple echocardiographic ratio of tricuspid annular metrics (such as systolic annular velocities s′ or plane systolic excursion TAPSE) to end‐systolic dimension would constitute a marker of ventriculo–arterial coupling. Compared with the SV/ESV ratio, integrating annular velocities and end‐systolic dimension into a single marker would offer the advantage to combine two well‐established echocardiographic prognostic factors in PH (Ghio et al., (2010); Amsallem, Sweatt, et al., 2017; Swift et al., 2017). To this end, we conducted a translational study first investigating the relationship between ventriculo–arterial coupling and a ratiometric index combining annular excursion peak velocity to RV end‐systolic area index (s′/RVESAi) in a validated large animal model of precapillary PH (Boulate et al., 2017; Mercier et al., 2013; Noly et al., 2015), and then determining its prognostic value in patients with pulmonary arterial hypertension (PAH).

The first objective was to compare the relationship between noninvasive echocardiographic RV metrics (including the s′/RVESAi ratio) and invasive ventricular contractility or ventriculo–arterial coupling in the piglet PH model, at baseline and after modulation of loading and contractility. The second objective was to validate s′/RVESAi in another piglet model of PH with severe pressure‐overloaded right ventricle. The third objective was to assess the prognostic value of s′/RVESAi for prediction of long‐term outcomes in two cohorts of patients with PAH: a first cohort of stable outpatients and a second cohort of acutely decompensated symptomatic patients.

## 2 METHODS

### 2.1 Animal models

Marie Lannelongue Hospital institutional animal care committee approved all procedures that were performed according to institutional guidelines complying with national and international regulations.

**Figure 1:**
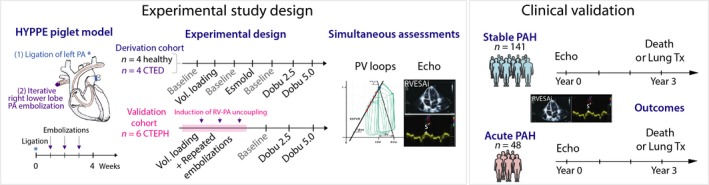
Experimental and clinical study design. CTED, chronic thromboembolic disease; CTEH, chronic thromboembolic hypertension; Dobu, dobutamine; PA, pulmonary artery; PAH, pulmonary arterial hypertension; PV, pressure–volume loops; RVESAi, right ventricular end‐systolic area index; Vol. loading, volume loading

All the experimental procedures and data acquisitions were performed under general anesthesia as previously described (Boulate et al., 2017, 2015). The derivation cohort included 8 large white pigs (*sus scrofa*) of 6‐week‐old at time of study enrollment divided into two groups: a first group of four healthy pigs (controls) and a second group of four pigs in whom mild PH was induced by performing an extra‐pericardial left pulmonary artery ligation followed by right lower lobe embolizations with embucrylate (Histoacryl^®^, B Braun, Medical) once a week for 3 weeks, as previously described and illustrated in Figure 1 (Mercier et al., 2013). Modulations of RV–PA coupling were performed using betablocker (esmolol) or beta‐agonist (dobutamine) infusion. Each animal underwent right PV‐loop acquisition and echocardiography at baseline (“baseline”), during infusion of esmolol at 500 μg kg^−1^ min^−1^, after esmolol infusion cessation (“second stable baseline” before dobutamine infusion), during 2.5 μg kg^−1^ min^−1^ of dobutamine infusion, and then during 5 μg kg^−1^ min^−1^ of dobutamine infusion. We also evaluated animals at baseline and after volume loading of 500 ml of saline in order to explore changes with volume loading. A period of 15 min of hemodynamic stabilization was observed during each condition prior to data acquisition.

The validation cohort included six additional pigs with acute severe PH on chronic thromboembolic pulmonary hypertension, in order to validate echocardiographic surrogates of RV–PA coupling in a severe setting (Figure 1). Acute changes in Ees/Ea and echocardiographic indices were assessed following hemodynamic compromise provoked by both acute volume and pressure overload, followed by hemodynamic stabilization using dobutamine, as previously published (Boulate et al., 2017). Briefly, RV–PA uncoupling was induced in animals with CTEPH by acute volume loading (60 ml/kg of saline infusion) followed by iterative pulmonary embolizations (bolus of 0.15 ml of embucrylate) until the systemic systolic arterial pressure dropped under 90 mmHg or the pulmonary to systemic systolic pressure ratio reached 0.9. After chronic PH was aggravated to acute PH, dobutamine was infused at 2.5 μg kg^−1^ min^−1^ and 5 μg kg^−1^ min^−1^. RV–PVloops and echocardiographic data were analyzed following the same methodology as in the derivation cohort.

### 2.2 Multibeat pressure–volume loops

The protocols of animal preparation, anesthetic maintenance, and mechanical ventilation have been previously reported (Guihaire et al., 2013; Noly et al., 2015). Briefly, a conductance catheter including a micromanometer (Ventri‐Cath^™^, Millar Instrument) was positioned into the right ventricle through a jugular vein under fluoroscopic control for PV loops acquisitions. Pressures and ECG signals were amplified and transmitted to a Powerlab 16/35 (AD instrument Pty Ltd) and continuously recorded at a rate of 1,000 Hz using LabChart 7 pro (v7.3.7 AD instrument, Pty Ltd). The conductance catheter was calibrated for pressure, volume, and blood electric resistivity (Rho cuvette) with the MPVS ultra^®^ system according to the manufacturer instructions. Parallel volume conductance was determined by infusion of 10 ml of 10% saline serum repeated thrice. The alpha calibration of stroke volume was performed at each dataset acquisition using a thermodilution method (Swan‐Ganz catheter, Edwards Lifesciences Corporation). The balloon for inferior vena cava occlusion was positioned at the level of the diaphragm under fluoroscopic control through a transfemoral approach. Inferior vena cava occlusions were repeated at least three times for each dataset acquisition. All acquisitions were performed during stable end‐expiration periods. Stroke volume was determined as the ratio of cardiac output over heart rate acquired by thermodilution methods, with three boluses per measurement. Total pulmonary resistance was calculated as the ratio of MPAP over cardiac output. Cardiac output and total pulmonary resistance were indexed to the body surface area (BSA) using the following formula 0.0734*weight(kg)^(2/3)^ (Swindle, Makin, Herron, Clubb, & Frazier, 2012).

The RV end‐systolic elastance (Ees) was determined as the slope of the linear regression of the end‐systolic pressure–volume relationship during inferior vena cava occlusion; the end‐systolic pressure–volume point corresponded to the ECG T wave. The pulmonary artery elastance (Ea) was the ratio of RV end‐systolic pressure over stroke volume; the RV–PA coupling was the Ees/Ea ratio. The end‐systolic pressure–volume relationship linear regressions was performed by the least square method using manually validated pressure and volume values from at least five cardiac cycles during inferior vena cava occlusion. Each Ees value was derived from the mean of two values from the analysis of two different inferior vena cava occlusions. For each regression, if the *R* ^2^ was <.90 or the difference between two values of the same set exceeded 10%, a third occlusion was analyzed and the two closest values were used. Ees and Ea were noted Eesi and Eai when indexed with BSA to take into account BSA variations between animals.

### 2.3 Echocardiography

Resting echocardiograms were acquired using a Vivid 9 console and a 5S probe (GE Healthcare), including RV focused apical 5‐chamber views, parasternal long‐ and short‐axis views. In pigs, the 5‐chamber view was used instead of the human 4‐chamber view as previously published (Guihaire et al., 2013). Each view was acquired during 3 beats during an end‐expiratory apnea, while the ECG signal was simultaneously acquired. Images were stored in cine loop format and analyzed offline on a commercially available GE EchoPAC workstation (GE Healthcare) by a cardiologist (MA) blinded for groups and hemodynamic results. RV dimensions were measured on the RV‐focused view and included: end‐diastolic (RVEDA) and end‐systolic areas (RVESA), indexed on the body surface area (RVEDAi, RVESAi). RV area change was defined as RVEDA‐RVESA. RV functional metrics included: maximal tricuspid velocity s′ using tissue Doppler imaging on the RV‐focused apical 5‐chamber view, TAPSE, RV fractional area change (defined as [RVEDA‐RVESA]/RVEDA). The isovolumic acceleration (IVA, a previously reported marker of ventricular contractility) of the tricuspid annulus was quantified from Tissue Doppler Imaging (TDI) acquisitions on the lateral tricuspid annulus (Vogel et al., 2002). In this study, the following ratios of annular indices divided by RV end‐systolic dimension were assessed: TAPSE/RVESAi, s′/RVESAi and RV area change/ RVESA (two‐dimensional surrogate of the Stroke volume/ESV ratio). RV free‐wall longitudinal strain produced less reliable results by tracking and was thus not included in the final analysis as the coefficient of variation was greater than 25%.

### 2.4 Clinical validation

Two validation cohorts of patients with PAH were included to evaluate the prognostic value of RV noninvasive metrics: stable patients (cohort 1) and patients admitted for acutely decompensated PAH (cohort 2). Stanford University Institutional Review Board approved the study, which was conducted in agreement with the Helsinki‐II declaration. All patients gave written informed consent.

The first cohort was selected from the previously published prospective Vera Moulton Wall Center Pulmonary Hypertension Registry at Stanford of patients with incident or prevalent idiopathic, familial, drug and toxins, or connective tissue disease‐related PAH (i.e., mean pulmonary arterial pressure ≥25 mmHg and pulmonary arterial wedge pressure ≤15 mmHg) (Amsallem, Sweatt, et al., 2017; Galiè et al., 2015). From the initial 228 patients, all 141 patients with available TDI of the tricuspid lateral annulus data at the time of enrollment were included. The second cohort included all 48 patients with tricuspid TDI available among a cohort of 85 patients with acutely decompensated symptomatic PAH (NYHA class II or more) previously published (Amsallem, Boulate, et al., 2017). All patients underwent resting echocardiography as previously described (Amsallem, Boulate, et al., 2017; Amsallem, Sweatt, et al., 2017); RVESAi and s′ were measured from the RV‐focused apical 4‐chamber view.

The end point was all‐cause mortality or need for lung transplantation during follow‐up in the two clinical cohorts. Death was verified by an independent investigator through the Stanford Pulmonary Hypertension database and the National Social Security Death Index; transplantation was verified through chart review. Patients who underwent transplantation were censored at the time of surgery.

### 2.5 Statistics analysis

Quantitative variables are expressed as median and interquartile range and compared using Mann–Whitney test if nonparametric, or mean and standard deviation (*SD*) if parametric (as assessed using the Kolmogorov–Smirnov test). Qualitative variables are expressed as number and percentage, and compared using Chi‐square test. Linear correlations between two quantitative variables are expressed using Pearson correlation coefficients (*R* ^2^), slopes, Y intercepts absolute values and 95% confidence intervals. RV metrics were remeasured blindly by a second observer (DB) in the derivation piglet cohort to determine the inter‐observer variability (assessed using Bland–Altman test). Cox proportional hazard models were used to determine correlates of outcome in the clinical cohorts; hazard ratios were normalized for one *SD*. Results were considered significant when two‐sided *p*‐values were <.05. Statistical analysis was performed using SPSS^®^ statistical software (SPSS V.19, Inc) or GraphPad Prism 6 (GraphPad Software, Inc.).

## 3 RESULTS

### 3.1 Derivation piglet cohort

**Figure 2:**
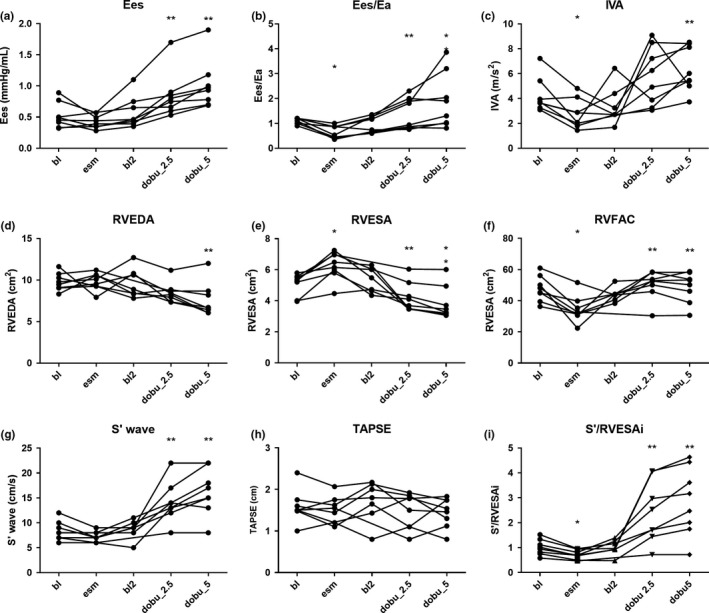
Changes in physiologic and echocardiographic parameters with betablocker (esmolol 500 μg kg^−1^ min^−1^) and dobutamine (2.5 and 5 μg kg^−1^ min^−1^) in the derivation cohort. (a) Ees, right ventricular end‐systolic elastance (b) Ees/Ea; Ea, pulmonary arterial elastance; (c) IVA, isovolumic acceleration of the tricuspid annulus; (d) RVEDA, right ventricular end‐diastolic area; (e) RVESA, right ventricular end‐systolic area (not indexed as no changes in body surface area are expected during acute changes of contractility and volume loading); (f) RVFAC, right ventricular fractional area change; (g) s′ wave; (h) TAPSE, tricuspid annular plane systolic excursion. (i) S'/RVESAi, ratio S'wave over right ventricular end‐systolic area index. *p < .05 versus baseline; **p < .05 versus baseline 2

The baseline characteristics of the eight pigs are presented in Table 1. Figure 2 and Table 2 present the changes in right heart metrics following esmolol and dobutamine infusion. Among echocardiographic indices, RVESA significantly increased with esmolol, while IVA and RVFAC significantly decreased (all *p* < .05). In contrast, s′ wave and TAPSE did not significantly change with esmolol. With a low dose of dobutamine (2.5 μg kg^−1^ min^−1^), cardiac output, heart rate, Ees/Ea and the three indices of RV contractility (Eesi, PRSW and +d*P*/d*t*) significantly improved (all *p* < .05). Among echocardiographic indices, RVESA decreased in size while RVFAC and s′ increased (all *p* < .05). With higher dose of dobutamine (5 μg kg^−1^ min^−1^), IVA and TAPSE also significantly increased (all *p* < .05). The noninvasive ratio s′/RVESAi was the only one that significantly decreased with esmolol and significantly increased with both dobutamine doses (all *p* < .05).

**Table 1:** Baseline characteristics of the derivation piglet cohort

	Animal model	Weight (kg)	MPAP at rest (mmHg)	Right atrial pressure (mmHg)	Cardiac output (L/min)	TPR (WU)	Ees/Ea ratio
1	Control	25	14	1	3.1	4.5	1.1
2	Control	26	9	1	2.4	3.8	1.2
3	Control	26	10	1	3.4	2.9	1.2
4	Control	26	11	2	4.3	2.6	1.2
5	CTED	22	23	5	2.3	10	1.0
6	CTED	24	24	3	3.2	7.5	1.2
7	CTED	20	17	5	2.5	6.8	1.0
8	CTED	18	16	4	2.2	7.3	0.9
Median [25th–75th]		25 [21–26]	15 [10.3–21.5]	2.5 [1.0–4.8]	2.8 [2.3–3.4]	5.7 [3.1–7.5]	1.15 [1.0–1.2]

Abbreviations: CTED, pigs with chronic thromboembolic disease but mean pulmonary artery pressure (MPAP) < 25mmHg; Ea, pulmonary arterial elastance; Ees, ventricular elastance; TPR, total pulmonary resistance.

**Table 2:** Variations of right ventricular metrics induced by esmolol and dobutamine in the derivation piglet cohort (*n* = 8)

	Baseline	Esmolol 500 μg kg^−1^ min^−1^	p*	Baseline 2	Dobu 2.5 μg kg^−1^ min^−1^	Dobu 5 μg kg^−1^ min^−1^	p**	p***
Heart rate (/min)	90 [77–101]	81 [73–93]	**0.047**	93 [78–103]	127 [110–134]	147 [124–153]	**0.008**	**0.007**
Right heart catheterization								
Stroke volume (ml)	36.0 [32.0–40.0]	30.0 [29.0–37.0]	0.259	37.5 [34.3–42.0]	38.0 [34.5–40.0]	38.5 [35.3–42.5]	0.798	0.645
Cardiac output (L/min)	2.8 [2.3–3.4]	2.5 [1.8–3.3]	**0.031**	3.5 [2.5–3.8]	4.7 [4.0–5.3]	5.4 [4.4–6.6]	**0.008**	**0.008**
MPAP (mmHg)	18 [17–19]	18 [17–21]	0.500	20 [17–24]	25 [20–26]	27 [21–31]	**0.008**	**0.008**
Ees/Ea	0.88 [0.71–1.13]	0.53 [0.41–0.87]	**0.031**	0.96 [0.64–1.26]	1.38 [0.84–1.97]	1.58 [1.01–2.87]	**0.008**	**0.008**
Eesi (mmHg/ml/m^2^)	0.31 [0.24–0.33]	0.25 [0.17–0.28]	0.078	0.29 [0.24–0.41]	0.44 [0.37–0.57]	0.55 [0.44–0.72]	**0.008**	**0.008**
Eai (mmHg/ml/m^2^)	0.27 [0.26–0.33]	0.41 [0.29–0;62]	**0.031**	0.34 [0.22–0.51]	0.39 [0.26–0.53]	0.36 [0.24–0.56]	**0.367**	**0.461**
PRSW (mmHg)	13 [9–16]	11 [9–14]	0.156	13 [12–15]	18 [15–25]	26 [17–29]	**0.016**	**0.008**
+d*P*/d*t* (mmHg/s)	371 [284–560]	337 [249–364]	**0.047**	360 [300–385]	667 [502–740]	1,020 [822–1139]	**0.008**	**0.008**
Echocardiography								
RVESAi (cm^2^/m^2^)	8.5 [7.9–9.2]	10.8 [9.1–12.1]	**0.008**	9.0 [8.0–10.2]	6.4 [5.5–8.8]	5.7 [5.0–8.1]	**0.039**	**0.016**
IVA (m/s^2^)	3.7 [3.4–5.1]	2.4 [1.9–3.8]	**0.016**	2.7 [2.7–4.4]	5.6 [3.4–8.2]	5.7 [5.1–8.3]	0.109	**0.030**
TAPSE (mm)	15[15–17]	15 [12–17]	0.313	17 [14–18]	16 [11–18]	15 [11–18]	0.469	0.578
s′ wave(cm/s)	8.0 [7.0–9.8]	7.0 [6.3–8.0]	0.125	9.0 [8.0–10.0]	14.0 [12.3–20.8]	16.0 [13.5–21.0]	**0.016**	**0.016**
RVFAC (%)	47 [40–55]	32 [31–39]	**0.008**	44 [41–44]	53 [47–57]	52 [41–57]	**0.016**	**0.047**
RV area change/RVESA	0.88 [0.69–1.21]	0.58 [0.45–0.81]	0.050	0.79[0.68–0.80]	1.12[0.89–1.34]	1.07[0.69–1.32]	**0.029**	0.152
s′/RVESAi (s^−1^·cm^−1^·m^−2^)	0.98 [0.77–1.28]	0.69 [0.54–0.91]	**0.008**	0.96 [0.92–1.26]	2.13 [1.51–3.78]	2.82 [1.81–4.23]	**0.016**	**0.016**
TAPSE/RVESA (cm^−1^)	0.28 [0.21–0.38]	0.23 [0.21–0.32]	0.094	0.29[0.18–0.45]	0.36[0.29–0.44]	0.38[0.18–0.51]	0.19	0.844

Note

Data are presented as median and interquartile range. Bold values indicate *p* < .05. *p‐value between baseline and esmolol. **p‐Value between baseline 2 (pre‐dobu) and dobutamine 2.5. ***p‐Value between baseline 2 (pre‐dobu) and dobutamine 5.

Abbreviations: Dobu 2.5, Dobutamine 2.5 μg kg^−1^ min^−1^; Dobu 5, Dobutamine 5μg kg min^−1^; Ea, pulmonary artery elastance; Ees/Ea, ventriculo–arterial coupling; Eesi, right ventricular end‐systolic elastance/body surface area; IVA, isovolumic acceleration of the tricuspid annulus; MPAP, mean pulmonary artery pressure; PRSW, preload recruitable stroke work; RVESAi, right ventricular end‐systolic area indexed on body surface area; RVFAC, right ventricular fractional area change; TAPSE, tricuspid annular plane systolic excursion;

### 3.2 Correlations between Ees/Ea, Eesi, and surrogates

**Figure 3:**
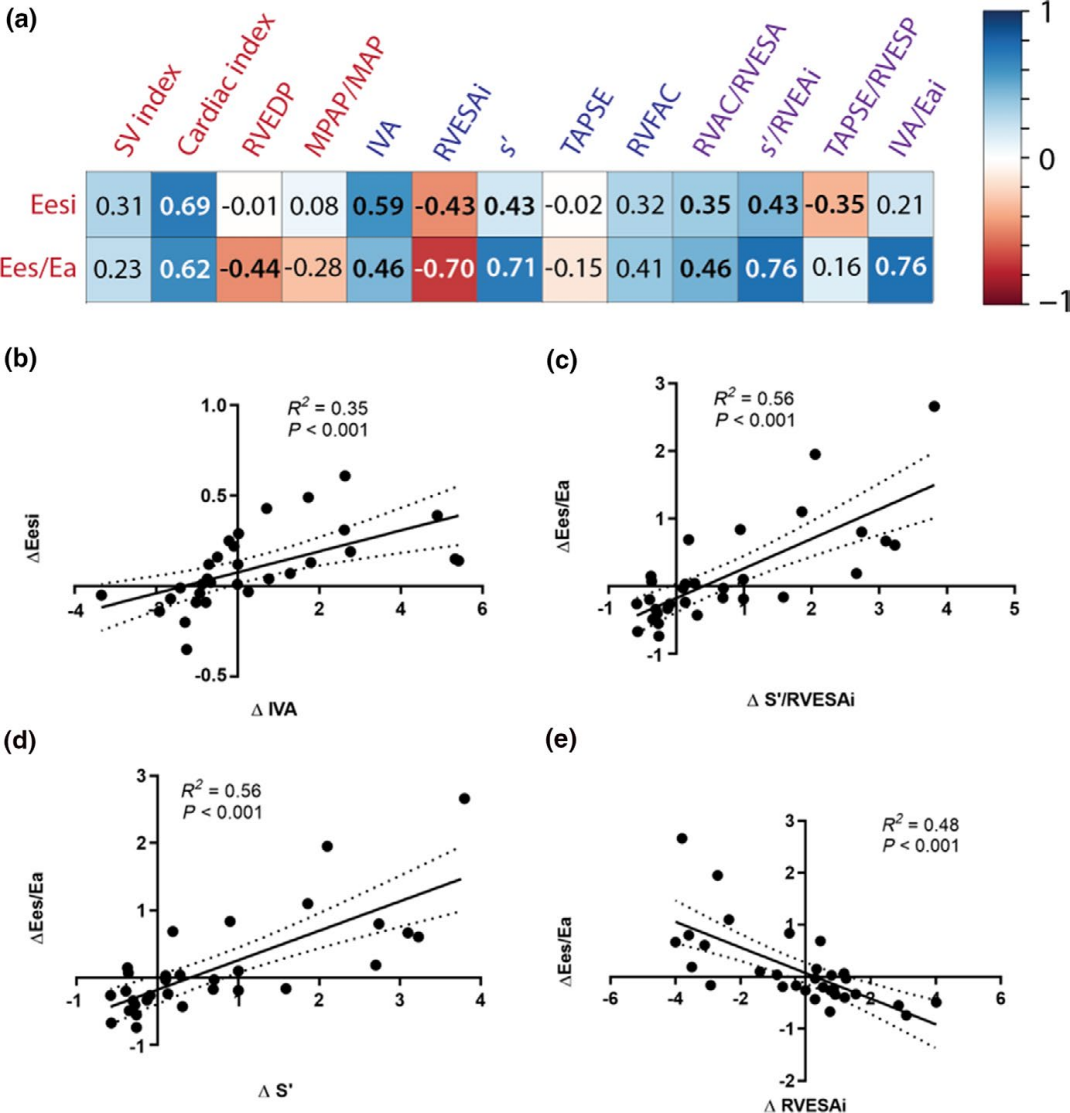
Correlations between right heart metrics values and variations in the derivation cohort. (a) Correlation heatmap of invasive (red), echocardiographic (blue), and ratios values. Correlations are presented using Pearson's correlation coefficients; significant correlations (with *p* values < .05) are presented as bold. The color bar (1 to −1) corresponds to correlation coefficients (*R* ^2^) (b–e). Correlations between changes in right heart metrics in the derivation cohort. Δ, variation of absolute values from baseline. Eesi, right ventricular end‐systolic elastance index, indexed for body surface area; Ees/Ea, RV–PA coupling, that is, right ventricular end‐systolic elastance/pulmonary arterial elastance); SV, stroke volume; RVEDP, right ventricular end‐diastolic pressure; MPAP, mean pulmonary artery pressure; MAP, mean pulmonary artery pressure; IVA, isovolumic acceleration of the tricuspid annulus; RVESAi, right ventricular end‐diastolic area index; s′, s′ wave; TAPSE, tricuspid annular plane systolic excursion; RVFAC, right ventricular fractional area change; RVAC, right ventricular area change; RVESP, right ventricular end‐systolic pressure; Eai, pulmonary arterial elastance index; Dashed lines represent 95% confidence interval

The Figure 3a illustrates the correlations between Eesi or Ees/Ea ratio and noninvasive metrics and ratios in the derivation cohort. The best correlates of Eesi among simple echocardiographic were IVA, RVESAi and s′, and among ratios s′/RVESAi. The best correlates of Ees/Ea among simple echocardiographic were also IVA, RVESAi and s′, and among ratio s′/RVESAi and the surrogate IVA/Eai. TAPSE/RVESA correlated poorly with Ees/Ea (*R* ^2^ = .11, *p* = .04) and did not correlate with Eesi (*R* ^2^ = .04, *p* = NS). The significant correlations between absolute value changes of s′/RVESAi, IVA and Ees/Ea are also presented in Figure 3b–e.

### 3.3 **Effect of volume loading on Ees/Ea and s**′**/RVESAi**

Volume loading of 500 ml of saline did not induce significant changes in Ees/Ea or s′/RVESAi in the derivation cohort, as shown in Figure S2 [https://figshare.com/articles/Figure_S2/9946883].

### 3.4 Interobserver variability

There was a good reproducibility of s′/RVESAi measurement between the two readers (Figure S3 [https://figshare.com/articles/Figure_S3/9946892]) with a correlation of *R* ^2^ = .94 (*p* < .01). The mean difference (standard deviation, [95% limit of agreement]) of s′/RVESAi between the two readers was −0.08 (0.31, [−0.68; 0.52]), of s′ was −0.02 (0.02, [−0.03; 0.04]) and of RVESAi was −0.23 (1.57, [−2.83; 3.30]).

### 3.5 Validation piglet cohort

**Figure 4:**
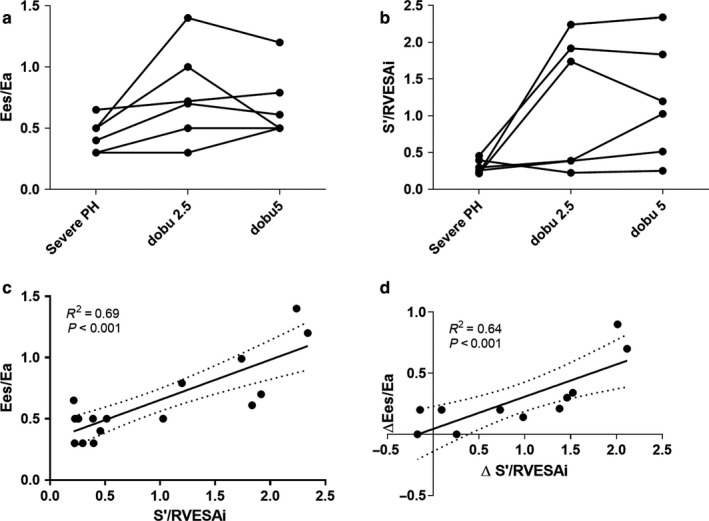
Validation of the value of s′/RVESAi in the validation cohort with severe pulmonary hypertension (*n* = 6). (a) Variations of Ees/Ea ratio and (b) the s′/RVESAi ratio at baseline (severe pulmonary hypertension), and after infusion of 2.5 and 5 μg kg^−1^ min^−1^ of dobutamine. (c) Relation between absolute values of s′/RVESAi and Ees/Ea, and (d) between their changes after infusion of 2.5 and 5 μg kg^−1^ min^−1^ of dobutamine. Dashed lines represent 95% confidence interval

The value of s′/RVESAi was then assessed in the validation cohort of six pigs with more severe PH than the derivation cohort animals. Baseline weight was 36 [28–37] kg and BSA was 0.80 [0.68–0.82] m^2^. Table 3 presents the hemodynamic and imaging characteristics at baseline (median MPAP of 50 [46–61] mmHg) and after RV–PA coupling restoration with dobutamine. After dobutamine infusion in the context of RV–PA uncoupling secondary to severe pressure overload, both s′/RVESAi absolute value and variation were significantly correlated with Ees/Ea absolute value and variation of absolute values (as illustrated in Figure 4).

**Table 3:** Characteristics of the validation piglet cohort (*n* = 6 with severe chronic pulmonary hypertension) at baseline and after dobutamine infusion

	Baseline severe acute on chronic PH	Dobutamine 2.5 μg kg^−1^ min^−1^	Dobutamine 5 μg kg^−1^ min^−1^
MPAP (mmHg)	50 [46–61]	74 [60–80]	75 [66–81]
Mean arterial pressure (mmHg)	66 [50–77]	94 [76–119]*	80 [75–115]
RV end‐diastolic pressure (mmHg)	16 [12–19]	12 [10–20]	18 [10–19]
Stroke volume (ml)	31 [30–40]	43 [37–50]*	44 [34–54]*
Heart rate (/min)	84 [75–107]	123 [102–130]*	136 [114–145]*
Cardiac output (ml/min)	3.1 [2.4–4.0]	4.7 [4.2–6.2]*	5.0 [4.5–8.0]*
Eesi (mmHg/ml/m^2^)	0.57 [0.46–0.75]	1.02 [0.66–1.88]*	0.93 [0.65–1.12]
RVESAi (cm^2^/m^2^)	16.0 [14.4–17.3]	9.9 [7.4–11.9]	11.3 [9.8–16.8]
s′ wave (cm/s)	4.5 [4.0–5.5]	14.0 [3.5–17.0]	12.5 [8.5–17.8]
Ees/Ea	0.45 [0.30–0.54]	0.71 [0.45–1.1]	0.55 [0.50–0.90]
s′/RVESAi (s^−1^·cm^−1^·m^−2^)	0.28 [0.22–0.41]	1.17 [0.30–2.08]	1.11 [0.45–1.96]

Note

Data are presented as median and interquartile range. *p < .05 compared with baseline value.

Abbreviations: Ees/Ea, right ventricular elastance/pulmonary artery elastance ratio; Eesi, right ventricular end‐systolic elastance indexed for body surface area; MPAP, mean pulmonary artery pressure; PH, pulmonary hypertension; RV, right ventricular; RVESAi, right ventricular end‐systolic area index.

### 3.6 **Clinical evaluation of s**′**, RVESAi and s**′**/RVESAi**

Baseline characteristics of the two clinical cohorts are presented in the Table S1 (https://figshare.com/articles/Table_1S/9946907). The median age was similar in the two groups (49 and 44 year, respectively), with a majority (80%) of female sex. The “acute” cohort (*n* = 48) was slightly more severe than the “stable” cohort (*n* = 141) as illustrated by a trend toward higher pulmonary vascular resistance (12.3 [9.7; 17.3] vs. 10.6 [7.2; 15.2]), lower cardiac index (1.84 [1.65; 2.23] vs. 2.03 [1.67; 2.35]) and higher NT‐proBNP levels. The mean (±standard error) survival rates were 92.0 ± 2.3% at 1 year and 79.3 ± 3.6% at 3 years for the “stable” cohort and 93.6 ± 3.6% at 1 year and 78.7 ± 6.0% at 3 years for the “acute” cohort. Using univariable analysis (Table 4), s′/RVESAi was a strong predictor of outcomes in both cohorts (hazard ratio per standard deviation = 2.64 [1.51–4.68] in the “stable” cohort and 2.89 [1.05–8.27] in the “acute” cohort). RVESAi was also associated with the risk of death or need for lung transplant in both cohorts (1.76 [1.37–2.24] and 2.23 [1.37–3.74], respectively).

**Table 4:** Univariate Cox regression analysis of correlates of death or lung transplant in the two cohorts of patients with pulmonary arterial hypertension

Variables	“Acute” cohort (*n* = 48)	“Acute” cohort (*n* = 48)	“Acute” cohort (*n* = 48)	“Acute” cohort (*n* = 48)		
	HR^a^	95% CI	*p*	HR^a^	95% CI	*p*
s′/RVESAi per *SD* decrease	2.64	1.51–4.68	**<.001**	2.89	1.05–8.27	**.04**
s′ per *SD* decrease	1.01	0.70–1.45	.95	1.11	0.65–1.90	.68
RVESAi per *SD* increase	1.76	1.37–2.24	**<.001**	2.23	1.37–3.74	**<.01**

Abbreviation: RVESAi, right ventricular end‐systolic area index.

a Hazard ratios are presented as HR per standard deviation (*SD*) for continuous variables, calculated as HR^SD^, with their 95% confidence interval (95% CI).

Bold values indicate *p* < .05.

## 4 DISCUSSION

The original contribution of our study is to quantify the association between commonly used noninvasive indices of RV function and ventricular arterial coupling in a large animal model of PH. From our experimental data, we confirm that commonly used indices of ventricular function relate more to ventriculo–arterial coupling than to ventricular elastance. Despite the small experimental size, the s′/RVESAi had the stronger nominal association with ventriculo–arterial coupling, and was the only metric that decreased significantly with esmolol infusion. In the clinical cohort, the point estimate for prediction of outcome of s′/RVESAi was higher than RV end‐systolic dimension alone, but its incremental value in clinical practice will likely be small. The index may, however, play a role in assessing acute changes of ventriculo–arterial coupling at bed side.

Few studies have analyzed noninvasive metrics of RV–PA coupling in large animal models of PH. The study of Vogel et al. validating IVA as a promising index of RV contractility was conducted in healthy pigs (Vogel et al., 2002). Here, we confirmed that IVA correlated with Eesi also in precapillary PH setting. Previous large animal PH models have primarily used air emboli (Zhou et al., 2011), pulmonary vein ligature (Garcia‐Alvarez et al., 2015) or distal pulmonary artery emboli (Kim et al., 2000). In contrast to these previous models, our piglet model has the two advantages of being evaluable through a large range of pulmonary artery pressures with both PV loops and echocardiography, and of reproducing chronic thromboembolic pulmonary hypertension pathophysiology.

Because the gold standard for Ees/Ea determination requires invasive PV loop conductance studies, efforts have focused on identifying surrogate end points. These often can be divided into three main categories. First, the single beat method measures ventricular elastance by extrapolating maximal RV pressure during isovolumic contraction and using the end‐systolic pressure–volume point derived from the single pressure volume loop (Brimioulle et al., 2003; Sunagawa et al., 1980) (Figure S1b). Second, the zero intercept end‐systolic method intercept (V0 method) is a method determining the end systolic pressure–volume relationship as the ratio of end‐systolic pressure/ end‐systolic volume assuming that V0 (right ventricular end‐systolic volume at RV end‐systolic pressure = 0 mmHg) is equal to 0 ml (Figure S1c). Third, combining several noninvasive echocardiographic indices such as the IVA and measures of load could also serve as surrogates for coupling. Although we validate the association between IVA and ventricular contractility (Ees) in our study, the use of IVA has not gained popular use in part due to reproducibility and potential intervendor methodological differences.

The main advantage of our study is to critically evaluate simple indices of RV–PA coupling. We first validate our previous observation that measures of systolic function mainly reflect RV–PA coupling and not right ventricular contractility but now across the spectrum of severity of PH (Guihaire et al., 2013). Building on this study, we demonstrate the strong association between RV end‐systolic dimension and RV ventriculo–arterial coupling. RV end‐systolic dimension is emerging as a strong predictor of outcome in PAH using both magnetic resonance imaging and echocardiography (Amsallem, Sweatt, et al., 2017; Swift et al., 2017). Our study further highlights the value of both annular velocities and end‐systolic dimensions. Our findings are supported by the baseline association between these indices and coupling, as well as with the changes with esmolol or dobutamine, confirmed in the validation piglet cohort.

The clinical implication of our study is physiological at this stage. As illustrated in our experimental section, the s′/RVESAi is a good metric of RV–PA coupling reflecting acute changes in volume loading and contractility. A potential application could be serial evaluation of RV adaptation to its afterload patients with PH, or, for example, for evaluation of the response to catecholamine infusion in patients in the intensive care unit or experiencing clinical worsening. The s′/RVESAi ratio has the clinical bedside strong advantage of being derived from a single echocardiographic view. Regarding the prognostic value, in our clinical cohorts, while the s′/RVESAi was strongly associated with outcomes, the confidence interval of its hazard ratio overlapped with the RVESAi′s, requiring further clinical validation to determine its potential incremental value.

The main limitation of our study is the angle‐dependency of s′ and RVESA measurements. Reproducibility of these metrics can be improved by using the RV‐focused apical view as previously reported (Amsallem et al., 2018). However, angle‐dependency limitation impact may be lowered by iterative evaluation and analysis of longitudinal values variations.

## 5 CONCLUSION

Our study validates using a large animal model of progressive RV pressure overload that annular indices as well as end‐systolic indices mainly reflect RV–PA coupling. While providing physiological basis for the use ratio of s′/RV end‐systolic dimension ratio, future studies will be needed to validate its incremental value in clinical practice.

## CONFLICT OF INTEREST

None of the authors have any conflict of interest relative to the study.
