# Supplementary figures and images for: Echocardiographic evaluations of right ventriculo–arterial coupling in experimental and clinical pulmonary hypertension
Source: Physiol Rep. 2019 Dec 25;7(24):e14322. doi: 10.14814/phy2.14322 (PMC6930934; doi:10.14814/phy2.14322)

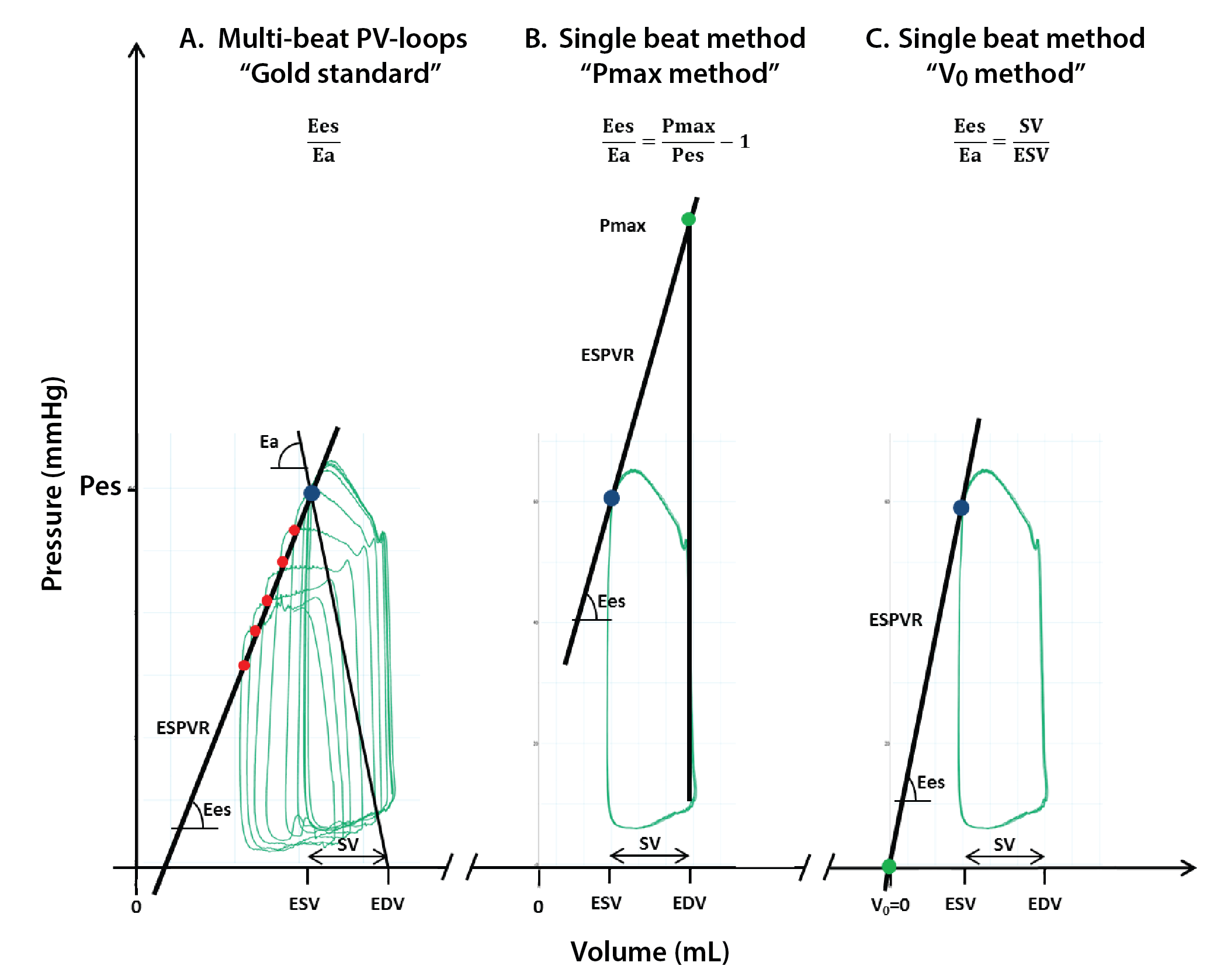

Supplement: Supplementary file 1 [file PHY2-7-e14322-s001.tif]
