# Supplementary material for: Echocardiographic evaluations of right ventriculo–arterial coupling in experimental and clinical pulmonary hypertension
Source: Physiol Rep. 2019 Dec 25;7(24):e14322. doi: 10.14814/phy2.14322 (PMC6930934; doi:10.14814/phy2.14322)
Supplement: Supplementary file 4 [file PHY2-7-e14322-s004.docx]

**Supplemental Table S1: Baseline characteristics of the two clinical validation cohorts of patients with pulmonary arterial hypertension (PAH).**

| **Variables** | **First cohort**  **n=141** | **Second cohort**  **n=48** |
| --- | --- | --- |
| Age (years) | 49.2 [38.2 ; 57.8] | 44.0 [37.0 ; 50.0] |
| Women | 113 (80.1) | 37 (77.1) |
| Body surface area (m²) | 1.84 [1.67 ; 1.99] | 1.83 [1.61 ; 1.96] |
| PAH Etiology  - Idiopathic or familial  - Drugs and toxins  - Connective tissue disease | 51 (36.2)  42 (29.8)  48 (34.0) | 17 (35.4)  23 (47.9)  8 (16.7) |
| New York Heart Association functional class | | |
| I | 9 (6.4) | 0 (0) |
| II | 59 (41.8) | 19 (39.6) |
| III | 59 (41.8) | 23 (47.9) |
| IV | 14 (9.9) | 5 (10.4) |
| **Hemodynamics** |  |  |
| Heart rate (bpm) | 80.0 [71.0 ; 86.5] | 78.0 [66.5 ; 86.5] |
| Systolic blood pressure (mmHg) | 114.0 [107.0 ; 126.0] | 117.0 [106.0 ; 131.0] |
| Right atrial pressure (mmHg) | 7.0 [5.0 ; 11.0] | 8.0 [5.0 ; 14.0] |
| Mean pulmonary arterial pressure (mmHg) | 51.5 [41.0 ; 59.8] | 55.0 [42.0 ; 64.0] |
| Pulmonary arterial wedge pressure (mmHg) | 10.0 [8.0 ; 13.0] | 10.0 [6.0 ; 13.0] |
| Cardiac Index (L/min/m^2^) | 2.03 [1.67 ; 2.35] | 1.84 [1.65 ; 2.23] |
| Pulmonary vascular resistance (WU) | 10.6 [7.2 ; 15.2] | 12.3 [9.7 ; 17.33] |
| Pulmonary vascular resistance indexed (WU.m^2^) | 18.7 [12.4 ; 26.0] | 23.4 [15.1 ; 30.7] |
| **Echocardiography** |  |  |
| RV end-diastolic area index (cm^2^/m^2^) | 18.0 [14.9 ; 22.1] | 18.1 [12.6 ; 20.9] |
| RV end-systolic area index (cm^2^/m^2^) | 12.8 [10.3 ; 16.5] | 13.7 [10.6 ; 16.9] |
| Right atrial area index (cm^2^/m^2^) | 11.4 [8.8 ; 14.9] | 13.4 [10.2 ; 17.0] |
| RV fractional area change (%) | 26.9 [22.9 ; 30.8] | 22.5 [19.1 ; 28.4] |
| RV free-wall longitudinal strain (%) | -16.4 [-20.5 ; -13.3] | -13.8 [-16.0 ; -11.0] |
| s’ maximal velocity (cm/s) | 11.2 [9.4 ; 13.1] | 11.5 [10.1 ; 13.0] |
| Tricuspid annular plane systolic excursion (mm) | 17.0 [14.0 ; 21.0] | 14.0 [12.0 ; 17.0] |
| s’/ RV end-systolic area index | 0.84 [0.62 ; 1.13] | 0.86 [0.68 ; 1.10] |
| RV systolic pressure (mmHg) | 79.5 [65.0 ; 93.8] | 81.0 [64.0 ; 99.6] |
| Right atrial pressure (mmHg) | 10.0 [5.0 – 15.0] | 10.0 [5.0 ; 15.0] |
| **Laboratory data** |  |  |
| Serum N-terminal pro B-type natriuretic peptide (pg/mL) | 288.0 [79.5 ; 1338.8] | 681.0 [303.0 ; 1646.0] |
| **PAH-specific therapy** |  |  |
| Prostanoid Therapy | 52 (36.9) | 16 (33.3) |
| Phosphodiesterase Inhibitors | 68 (48.2) | 22 (45.8) |
| Endothelin Receptor Blockers | 42 (29.8) | 13 (27.1) |

Data is presented as median [interquartile range] or number (percentage). RV: right ventricular.
